# Evaluation of whey protein coating containing free and Pickering emulsion forms of *Trachyspermum copticum L*. essential oil on quality of refrigerated beef

**DOI:** 10.1002/fsn3.4454

**Published:** 2024-09-28

**Authors:** Vida Saghari, Hossein Jalali, Nabi Shariatifar, Seyedhamidreza Ziaolhagh

**Affiliations:** ^1^ Department of Food Science and Technology, Damghan Branch Islamic Azad University Damghan Iran; ^2^ Department of Environmental Health, Food Safety Division, School of Public Health Tehran University of Medical Sciences Tehran Iran; ^3^ Drug Design and Development Research Center, the Institute of Pharmaceutical Sciences (TIPS) Tehran University of Medical Sciences Tehran Iran; ^4^ Agricultural Engineering Research Department Agricultural and Natural Resources Research Center of Semnan Province (Shahrood), AREEO Shahrood Iran

**Keywords:** antimicrobial properties, biodegradable coating, microbiological analysis, physicochemical analysis, sensory evaluation

## Abstract

The purpose of the present research was to prepare a novel biodegradable coating of whey protein (whey) and essential oil of *Trachyspermum copticum L.* (forms of free (EO) and Pickering emulsion (NEO)) to improve the shelf life of beef. In this study, various microbiological, chemical, and sensory analyses were performed. The results showed that after 18 days, the highest and lowest microbiological counts were related to control samples and whey‐NEO treatments, respectively. The total viable count (TVC) was 8.2 and 6.8 log CFU/g, the total psychrotrophic count (TPC) was 8.5 and 6.7 log CFU/g, and the lactic acid bacteria (LAB) count was 7.7 and 6.5 log CFU/g for control and whey‐NEO treatments, respectively. Chemical analysis of whey‐NEO treatment and control sample were 6.3 and 7.5 for pH, 9.8 and 13.9 meq/kg for PV (peroxide value), 5.45 and 8.85 mg/kg for TBARS (thiobarbituric acid reactive substances), and 25.1 and 39.4 mgN/100 g for TVB‐N (total volatile basic nitrogen), respectively. The best result was associated with the whey‐NEO treatment; consequently, coatings with whey and EO Pickering emulsion should be considered as a potential active coating in the meat industry.

## Graphical abstract

Our results showed that after 18 days, the highest and lowest microbiological counts were related to control samples and whey‐NEO treatments, respectively. The total viable count (TVC) was 8.2 and 6.8 log CFU/g, the total psychrotrophic count (TPC) was 8.5 and 6.7 log CFU/g, and the lactic acid bacteria (LAB) count was 7.7 and 6.5 log CFU/g for control and whey‐NEO treatments, respectively. Chemical analyses for control samples and whey‐NEO treatments included pH = 6.3 and 7.5 (), peroxide value (PV) = 9.8 and 13.9 meq/kg, thiobarbituric acid reactive substances (TBARS) = 5.45 and 8.8 mg MDA/kg, and total volatile basic nitrogen (TVB‐N) = 25.1 and 39.4 mg N/100 g, respectively. The best result was associated with the whey‐NEO treatment; consequently, coatings with whey and EO Pickering emulsion should be considered as a potential active coating in the meat industry.

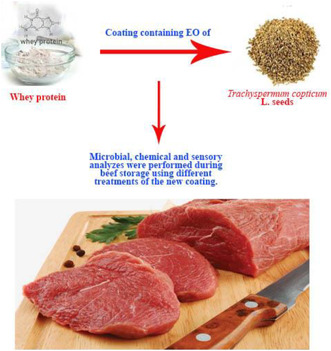

## 1 INTRODUCTION

The presence of microorganisms, chemical pollution, and other foreign substances in food can cause food spoilage, and as a result of this spoilage, food loses its nutritional value and edible quality, and as a result of consuming these foods, the health of consumers and society can be endangered. Recently, in order to increase food quality and reduce food safety risks during various processes, including production and storage, the use of packaging materials in the food industry has increased widely. However, packaging materials such as synthetic plastics and similar materials pose a significant threat to food safety, human health, and environmental pollution. For this purpose, the use of natural and biodegradable packaging materials, considering that they do not have the side effects of synthetic compounds and also have biodegradable properties, can be a suitable alternative (Shariatifar et al., 2019; Shariatifar et al., 2020; Soltani Howyzeh et al., 2018; Torabiardekani et al., 2023; Zomorodian et al., 2023).

Using plant extracts and essential oils or their compounds as natural preservatives can increase the quality and shelf life of food and control its microorganisms. Plant essential oils and types of plant secondary metabolites are known as substances with antimicrobial properties and antioxidant activity (Anvar et al., 2023; Ardekani et al., 2019; Bahrami et al., 2023; Kardan‐Yamchi et al., 2020).

Usually, the EO of plants contains at least 15–60 components with different concentrations, which include active compounds from the group of alcohols, phenols, terpenes, aldehydes, ketones, ethers, esters, and many other compounds. Essential oils, which are considered natural compounds, are preferable to synthetic chemical compounds due to their environmentally friendly properties and safe effects on the human body when used as medicine and food. The compounds commonly identified in EOs are of low molecular weight and can be classified into 2 separate groups: the first group includes aliphatic and aromatic compounds, while the second group is divided into terpenoids and terpenes. In essential oils, major components (2 or 3 compounds) present at high concentrations (20%–70% percent) govern the biological characteristics of the EOs (Cahyana et al., 2022; Dhifi et al., 2016).

*Trachyspermum copticum L*. is one of the annual plants with light green leaves and belongs to the parsley family. This plant contains compounds such as extract, essential oil, 39.8% moisture, 68.15% protein, 36% carbohydrate, 8.8% fiber, 8.9% ash, furan, and coumarin, as well as various polyphenols and minerals (such as sodium, magnesium, calcium, phosphorus, and potassium). *Trachyspermum copticum L*. is an annual plant that is widely planted in different parts of Iran, including the southern regions. In general, the leaves and seeds of *Trachyspermum copticum L*. are used as food seasoning. This plant has been used since ancient times to solve digestive problems of the digestive system. Its seeds are used in traditional medicine to treat jaundice and liver diseases. In addition, this plant has antioxidant effects and is good at inhibiting free radicals (Kardan‐Yamchi et al., 2020; Rasooli et al., 2008).

Whey protein is one of the by‐products of the cheese‐making process and is able to produce a favorable film with mechanical properties, high transparency and resistance, as well as good biodegradability. Researchers have investigated various additives such as flavors, antioxidant and antimicrobial agents, dyes, and spices to whey‐based biodegradable films (Bahram et al., 2014; Seydim & Sarikus, 2006).

An emulsion is a mixture in which small droplets of a liquid (such as oil) are dispersed without mixing with another liquid (such as water). A biphasic dispersion emulsion is an immiscible liquid in the presence of an emulsifier that reduces surface tension by absorbing amphiphilic molecules at the water–oil interface. Pickering emulsion is a nanoemulsion technique that can increase emulsion stability by using solid particles at the interface between the water and oil phases. Pickering emulsions are considered to be safe, biodegradable, and natural, so their applications in several fields, such as foodstuff, biomedicine, and cosmetics, are very promising, including as a means of preserving essential oils (EOs) (Cahyana et al., 2022; Zhou et al., 2018; Zomorodian et al., 2023).

One of the new technologies in the world is nanotechnology, which has created a huge revolution in the food and agriculture industry. The use of natural compounds, including essential oils, in the form of nanotechnology in the food industry increases the effect of natural compounds. Controlling the size of nanoscale particles may cause changes in the macroscopic properties of materials such as texture, flavor, sensory properties, processability, and food stability (Anvar et al., 2023; Cahyana et al., 2022; Kardan‐Yamchi et al., 2020; Zomorodian et al., 2023).

Beef is an important source of muscle protein, which has a higher biological value than other muscle proteins. In addition, meat has a large amount of amino acids and minerals that increase its nutritional value. In recent years, the consumption of meat and meat products has increased. Meat and its products provide sufficient amounts of nutrients. Due to the presence of various compounds, meat is susceptible to the growth of microorganisms, the increase of oxidation, and the accumulation of some undesirable compounds. Nowadays, due to the side effects of chemical preservatives, the demand for the use of natural antioxidant and antibacterial compounds in the food industry, such as the meat industry, has increased. Among natural additives, EOs and plant extracts are good sources of antioxidants and antimicrobials and have high potential for application in the field of green consumerism (Ben Hsouna et al., 2017; El Abed et al., 2014; Sirocchi et al., 2017).

Considering that beef is one of the most important protein foods with high nutritional value among the foods of the Iranian household and that no comprehensive research has been done on the proper storage of beef in Iran and the world, the current research seems very necessary. It should be noted that this study was conducted for the first time in Iran and the world in order to investigate the effect of coating containing Pickering emulsion of *Trachyspermum copticum L*. essential oil and whey protein on the protection of beef. Thus, the principal purpose of this study was to evaluate a coating with whey protein and *Trachyspermum copticum L*. EO (forms of Pickering emulsion and free) to protect beef against the microbial (TPC, LAB, and TVC), chemical (TVB‐N, pH, TBARs, and PV), and sensory (off‐odor, red color, texture, and discoloration) changes for 18 days storage at 4°C.

## 2 MATERIALS AND METHODS

### 2.1 Reagents

Whey powder with 80% (w/w) purity was purchased from Fonterra, Ltd. Company (Auckland, Australia). Ethanol, acetic acid, anhydrous sodium sulfate (Na_2_SO_4_), glycerol (>97% purity), sodium hydroxide (NaOH), Folin–Ciocalteu reagent, and zein were purchased from Merck Company (Darmstadt, Germany). Other applied reagents and solvents were of analytical grade or higher available purity and purchased from Merck Co.

### 2.2 Plant material and preparation of EO

*Trachyspermum copticum L*. seeds were purchased from the Molavi market of Tehran, Iran, and authenticated by a pharmacognosy specialist at Tehran University of Medical Sciences. Based on the previous study, *Trachyspermum copticum L*. seeds were washed with distilled water and dried at ambient temperature in the shade, then powdered and essential oil was extracted by an apparatus of all‐glass Clevenger‐type for 4 h (Pabast et al., 2018). The yield of EO was obtained by the Clevenger method (2.2%). The color of the EO was yellow. Finally, the prepared EOs were stored in sealed vials at 4°C until use.

### 2.3 The identify EO components by GC/MS

A gas chromatography/mass spectrometry (GC/MS) (Agilent 7890A, USA/5975C VL MSD with Triple‐Axis detector) device was used to identify the compounds in the essential oil of the plant. The conditions of GC–MS like column, temperature, time, etc. were according to our previous study (Pabast et al., 2018).

### 2.4 Preparation of *Trachyspermum copticum L*. EO Pickering emulsion

Zein protein (1 g) was dissolved in glacial acetic acid solution (70 mL, 15% v/v) at room temperature to form zein stock solutions. Then, the stock solutions of zein were added dropwise to deionized water (130 mL) and mixed with a homogenizer at 12,000 rpm at high speed for 10 min and then with an ultrasonic diffuser (model: XC‐5 L, Nanjing Ningkai Instrument Co., Ltd, China) under 450 watts and a frequency of 40 kHz to produce zein colloidal particles with small sizes. Finally, Pickering emulsion of *Trachyspermum copticum L*. was prepared by mixing zein suspension and essential oil at a concentration of 2% and then homogenized at 12,000 rpm for 10 min (Almasi et al., 2020; Shen et al., 2021; Sun et al., 2020).

### 2.5 Whey protein coating production

The first, whey powder (8 g) was mixed and dissolved in distilled water (100 mL), and by adding NaOH (1%), the solution's pH was set to 8. Then the mixture was heated at 80°C for 30 min (Asdagh et al., 2021).

### 2.6 Preparation of edible coating

For this purpose, whey (8%, w/v) was mixed and dissolved with acetic acid (1%, v/v) and glycerol (as plasticizer). The coating solution was mixed on a magnetic hotplate mixer at 350 rpm at 40°C for 3 h. Next, the coating solution (20 mL) was poured into the Petri dishes, and then EO and NEO were added to the mixture at the ratio of 0.1% and 5% (w/v), respectively, and kept for 48 h. The mixtures were used for the tests and coating of beef samples (Homayounpour et al., 2020; Pabast et al., 2018).

### 2.7 Particle size measurements

Particle size was assessed by a particle size analyzer (model Nano‐ZS90, Malvern, UK). Before the test, the emulsions were diluted 100 times with ultra‐pure water and agitated well. Each emulsion was measured in triplicate (Xu et al., 2020).

### 2.8 SEM (scanning electron microscopy) and TEM (transmission electron microscopy) analysis

In this research, SEM image was used to investigate the structure and morphology of the prepared coatings by SEM analyzer (KYKY‐EM 3200; KYKY Technology Development Ltd., Beijing, China) according to previous research (Homayonpour et al., 2021; Pabast et al., 2018). Moreover, a TEM microscope (Philips Bio‐Twin, the Netherlands) was applied to explore the prepared coatings characterization, according to the previous study (Homayonpour et al., 2021).

### 2.9 Samples preparation

Beef was obtained from Tehran market 48 h after slaughter. After that, using sterilized knives and cutting boards, 96 meat steaks (6 × 6 cm in size, 2 cm in thickness, and 60 g in weight) were aseptically cut. Before coating the said steaks by immersion method, the beef samples were placed in a polystyrene tray and kept in the refrigerator until treatment. After that, all samples were divided into 4 groups or treatment samples: (i) uncoated (control); (ii) coated with whey (whey); (iii) coated with whey and EO (whey‐EO); (iv) coated with whey and Pickering emulsion of EO (whey‐NEO) and each sample for 1 min was immersed in the corresponding solutions. The mentioned samples were dipped in the coating solutions for 1 min to achieve a coating layer on the surface of the meat samples. Next step, the prepared samples were allowed to drip additional solution. Then, all samples were dried at 25°C for 15 min. Finally, samples were placed into sterilized plastic Petri dishes (separately) and sealed (hermetically), and kept in the refrigerator at 4°C for up to 18 days. From each four mentioned treatments (control, whey, whey‐EO, and whey‐NEO), 3 samples were prepared and examined on 7 days (0, 3, 6, 9, 12, 15, and 18) (Homayounpour et al., 2020; Pabast et al., 2018).

### 2.10 Microbiological analysis (TPC, LAB, and TVC)

In this study, analyses of LAB and TVC were performed based on Homayounpour et al.'s research (Homayonpour et al., 2021). TPC analysis was performed after incubation at 4°C for 18 days, based on Mohan et al.'s research (Mohan et al., 2012). The results were expressed in log CFU/g form.

### 2.11 Chemical analysis (thiobarbituric acid reactive substances (TBARs), total volatile base (TVB‐N), peroxide value (PV), and pH value)

The TVB‐N test was performed based on the research of Tometri et al. (2020). The PV and the pH tests (digital pH meter, Company of HANNA, Germany) were performed based on the research of Homayonpour et al. (2021). Also, the TBARS test was performed based on the research of Pabast et al. (spectrophotometry, Ultrospec 2000, the UK) (Pabast et al., 2018). In this research for assessing TBARS value, beef (10 g) were homogenized for 2 min at 4000 rpm with 30 mL of perchloric acid (4%) and BHT (butylated hydroxytoluene) solution (1 mL) dissolved in ethanol. A Whatman filter (No. 4) was applied to filter the mixture before usage. In a stoppered test tube, the subsequent solution (5 mL) was mixed with TBA (5 mL, 0.02 M). As part of a water bath, the combination was kept for 60 min at 95°C before being cooled in freezing water for 5 min. At the end of this technique, groups of treatments and control were read at a wavelength of 532 nm with a spectrophotometer (Ultrospec 2000, the UK) using a mixture of perchloric acid (5 mL, 4%) and TBA (5 mL, 0.02 M) as a sample blank.

### 2.12 The evaluation of sensory

Sensory tests were evaluated according to the method of Pabast et al. (by six semi‐trained panelists, which had a history of assessing samples of meat) (Pabast et al., 2018). In our research, 4 sensory items were analyzed, including color, off‐odor, discoloration, and texture. Also, when the sensory characteristics were higher than the value of three, the sample was no longer of good quality and was rejected.

### 2.13 Statistical analysis

All attained data were revealed as means±SD. By the ANOVA statistical test (using SPSS V. 24), our outcomes were evaluated. Moreover, the significant differences by Duncan's multiple range statistical test were evaluated (at *p* < .05). All chemical, microbial and sensory analyses, were repeated three times.

## 3 RESULTS AND DISCUSSION

### 3.1 Analysis of essential oil compounds

In our study, 17 EO compounds (according to Table 1) constitute 97.9% of the identified compounds, among which the main identified components are thymol (42.03%), p‐cymene (22.27%), γ‐terpinene (15.36%), carvacrol (4.73%), β‐Pinene (3.97%), and octyl butyrate (3.1%).

**TABLE 1:** Investigation of *Trachyspermum copticum L*. EO compounds by GC/MS.

Peak no.	Compound	RT (min)	A%
1	α‐Thujene	10.08	0.47
2	α‐Pinene	11.23	0.43
3	Sabinene	11.41	1.32
4	β‐Pinene	13.25	3.97
5	β‐Myrcene	14.03	0.25
6	α‐Phyllanderene	14.67	0.07
7	α‐Terpinene	15.31	1.16
8	p‐cymene	15.93	22.27
9	β‐Phyllanderene	16.27	0.55
10	γ‐terpinene	17.33	15.36
11	α‐Terpinelene	18.37	0.09
12	α‐Terpineol	23.88	1. 1
13	Terpine‐4‐ol	27.82	1.57
14	Thymol	28.27	42.03
15	Carvacerole	29.33	4.73
16	Octyl butyrate	32.72	3.1
17	Apiole	41.35	0.53
Total			97.9

Our findings are somewhat similar to other studies, that minor differences in the quantity of these compounds can be due to weather conditions, soil type, and desired plant species (Kardan‐Yamchi et al., 2020; Rasooli et al., 2008; Soltani Howyzeh et al., 2018).

### 3.2 Particle size

Particle size is a chief index to indicate the stability and uniformity of the system of emulsion. The distribution of size becomes narrower as the mixing speed rises. It is stated that the particle size can have a strong impact on the Pickering emulsions' stability. The nanoparticles to be applied as stabilizer shouldn't be too large to prevent the emulsion's aggregation and not too small because they should be sufficiently strongly attached at the interface of oil–water, with a free energy of attachment that largely exceeds the thermal energy of particles (Wu et al., 2015). In this study, the mean ± SD particle size of the emulsion was 1.92 ± 0.6 μm, indicating that higher zein concentrations were requisite to decrease the size of the oil droplets and stabilize the emulsion. Our results were confirmed by Xu et al., who analyzed the particle size of clove essential oil Pickering emulsion and reported the particle size of this emulsion ranged from 1.73 μm to 1.40 μm (Xu et al., 2020).

### 3.3 SEM analysis

**FIGURE 1:**
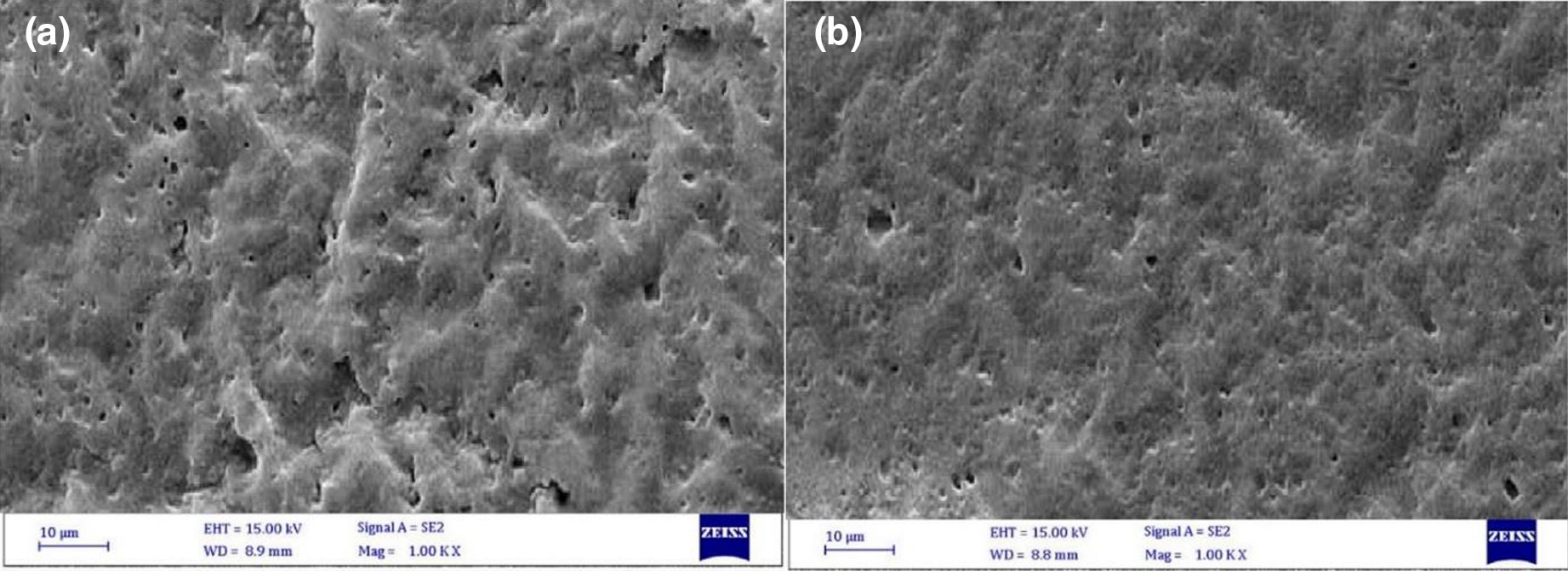
SEM image of whey (a) and whey‐NEO (b) coatings.

In our research, the surface morphology of whey and whey‐NEO coatings was evaluated by SEM analysis (Figure 1a,b). Using this micrograph, the homogeneity of the composite, the surface dispersion of nanoparticles in the sample matrix, the presence of empty space, the presence of mass, and in some cases the orientation of nanoparticles were detected. The micrograph of the whey coating showed that this coating is non‐empty, non‐porous, and smooth. Furthermore, the results (Figure 1a,b) showed that there was no large aggregation in whey‐NEO, which indicated the proper dispersion of whey‐NEO in the coating matrix. Our results were confirmed by other researchers (Li et al., 2022; Shahbazi et al., 2021; Sun et al., 2020).

### 3.4 TEM analysis

**FIGURE 2:**
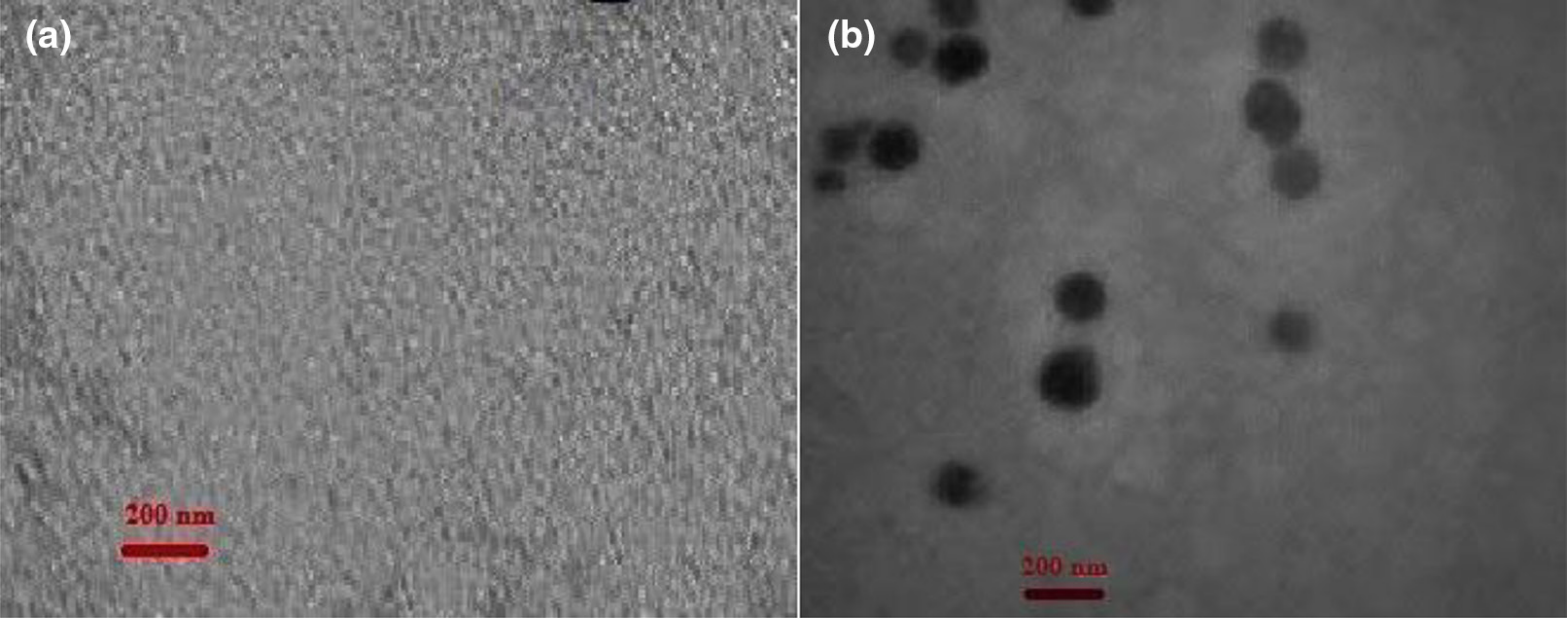
TEM of whey (a) and whey‐NEO (b) coatings.

Figure 2a,b shows a TEM image of whey (A) and whey‐NEO (B). The TEM image was accomplished to indicate the morphology of coatings, based on the prior studies (Pabast et al., 2018; Pouryousef et al., 2022a, 2022b). As exhibited in these figures, the structure of Pickering emulsion (nano) can be seen as dark circles on the surface of the film, which confirms the formation of Pickering emulsion (nano).

### 3.5 Microbial analysis

**FIGURE 3:**
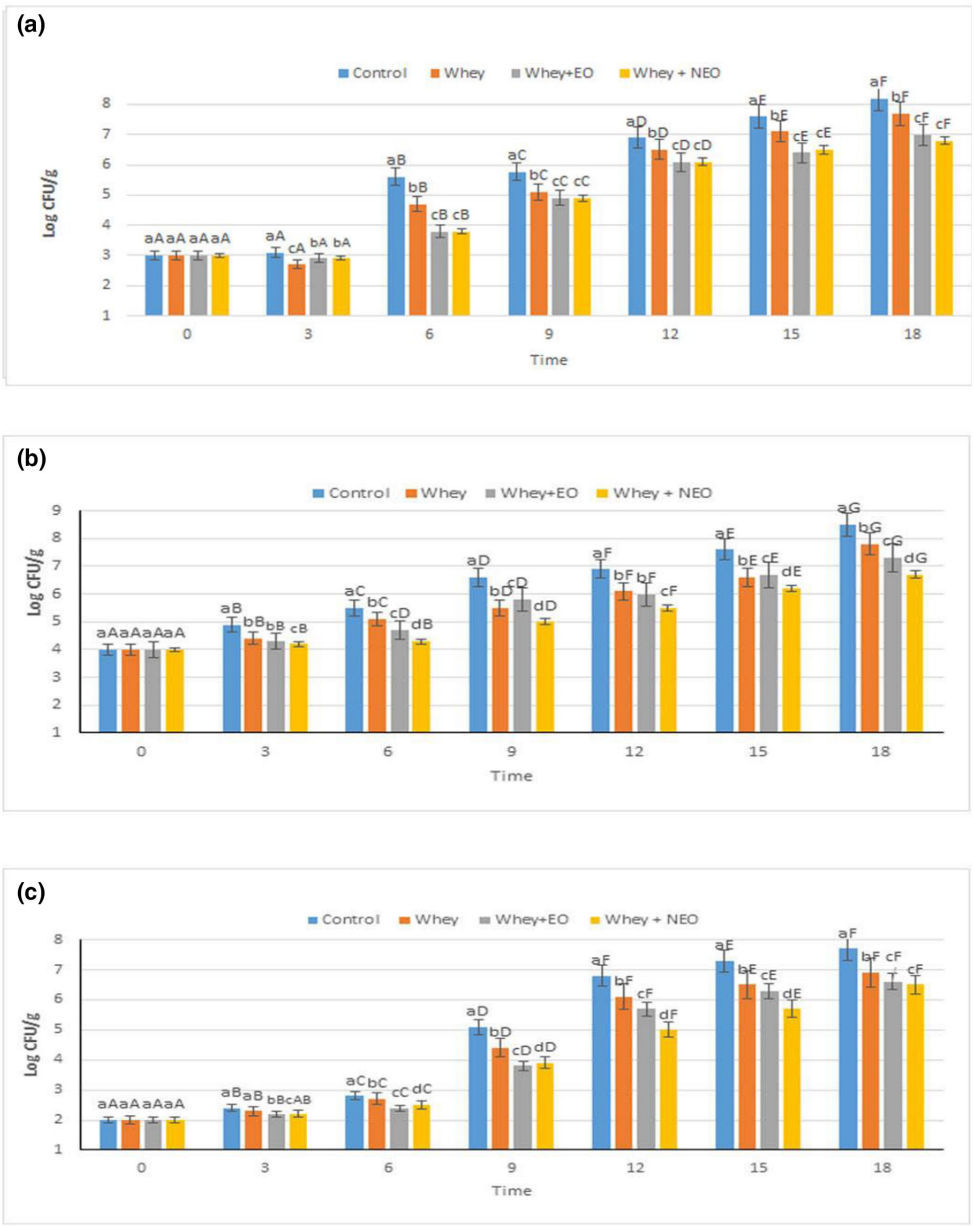
The TVC count of control and treatments storing for 18 days at 4°C (a), the TPC count of control and treatments storing for 18 days at 4°C (b), and the LAB count of control and treatments storing for 18 days at 4°C (c). In each group different lowercase letters are significantly different, *p* < .05; between group different superscript letters are significantly different, *p* < .05.

The quality of beef samples and their shelf‐life were evaluated by microbiological tests. In this study, TPC, LAB, and TVC analyses as microbial tests were performed during 18 days of storage at 4°C, which are shown in Figure 3.

#### 3.5.1 TVC

Figure 3a shows the TVC value of control samples and treatment samples (whey, whey‐EO, and whey‐NEO). The acceptable limit of TVC for fresh beef is 7 log CFU/g (Homayounpour et al., 2020; Pabast et al., 2018), and, for all samples, the TVC value on the zero‐day was between 2.7 and 3.1 log CFU/g, thereby showing their suitable conditions. After 15 days, the control and whey samples had exceeded 7 log CFU/g. After 18 days, all treatments and control samples were above 7 log CFU/g, except for whey‐NEO treatment. The minimum value of TVC was measured in whey‐EO (7 log CFU/g) and whey‐NEO treatment (6.8 log CFU/g) after 18 days, which may be due to the *Trachyspermum copticum L*. EO antibacterial characteristics (owing to the existence of polyphenols) that these compounds are effective against *Enterobacteriaceae*, mesophilic lactobacilli, *lactococcus*, aerobic mesophilic bacteria, yeasts, and enterococci (Radjabian et al., 2013). The antimicrobial nature of whey has been demonstrated by previous studies (Kanatt et al., 2013). Furthermore, Figure 3 reveals that the treatment of whey containing Pickering emulsion (whey‐NEO) raised the characteristics of the antimicrobial coating compared to the free form (whey‐EO), which is probably due to the greater protection of the compounds, the reduction of EO evaporation, the easier transfer to the cell wall of bacteria, and their encapsulation of the compounds in the nano‐emulsion (Homayonpour et al., 2021). The low microbial load in the form of Pickering emulsion has been confirmed by a previous study (Ghaderi‐Ghahfarokhi et al., 2017). The antimicrobial property of *Trachyspermum copticum L*. EO has been reported by other researchers (Kardan‐Yamchi et al., 2020; Rasooli et al., 2008). Also, Socaciu et al. analyzed the effects of coating containing tarragon EO and whey, which showed that after 15 days, the amount of TVC for the control and treatment samples varied from 6.68 to 8.65 log CFU/g, respectively (Socaciu et al., 2021). In addition, Zomorodian et al. investigated the effect of coating, including *Zataria multiflora* EO (forms of Pickering emulsion and free) and chitosan on the amount of TVC value of salmon fish and stated that the amount of TVC value in Pickering emulsion treatment was the lowest among other treatments and control samples, which confirmed our results (Zomorodian et al., 2023).

#### 3.5.2 TPC

Figure 3b shows the TPC value of control and treatment samples (whey, whey‐EO, and whey‐NEO) at 4°C for 18 days. The initial quality of the beef samples was good, and the microbial load was low (4 log CFU/g). During the experiment, the bacteria count (in control and treatment samples) showed an increasing trend. According to Figure 3b, after 18 days, the maximum bacterial load was detected in the control sample (8.5 log CFU/g) and the whey‐NEO treatment (6.7 log CFU/g) had the lowest bacterial load. These properties are typically attributed to the antibacterial effect of essential oil and whey (owing to the phenolic compounds and other antibacterial compounds), the interaction with anionic groups on the bacterial cell surface, lipopolysaccharide layer disruption of the outer membrane of bacteria, and function as a barrier against transmission of oxygen (Mohan et al., 2012). In the research of Socaciu et al., who analyzed the effects of coatings containing tarragon EO and whey during 15 days, the count of psychrotrophic control and treatments ranged from 7.49 to 8.57 log CFU/g, respectively (Socaciu et al., 2021). Also, the antimicrobial property of *Trachyspermum copticum L*. EO has been stated by other researchers (Kardan‐Yamchi et al., 2020; Rasooli et al., 2008).

#### 3.5.3 Lab

Figure 3c exhibits the value of LAB during 18 days of storage at 4°C for control and treatment samples (whey, whey‐EO, and whey‐NEO). The beef samples initial quality was proper, and a load of microbials was low (2 log CFU/g). During the experiment, the bacterial count showed an increasing trend (in control and all treatment samples). Based on Figure 3c, after 18 days, the minimum and maximum LAB counts were revealed in the whey‐NEO (6.5 log CFU/g) and control (7.7 log CFU/g) samples, respectively. Our mentioned treatments prevent the growth of bacteria due to the polycationic nature of whey and EO and disrupting the bacterial cell membrane, which other researchers have also confirmed (Kanatt et al., 2013) (Pabast et al., 2018). The antimicrobial and antibacterial properties of *Trachyspermum copticum L*. EO have been confirmed by other studies (Kardan‐Yamchi et al., 2020; Rasooli et al., 2008). In the research of Socaciu et al., the effects of coating containing tarragon essential oil and whey were investigated; they expressed the count of LAB after 15 days for control, and the treatment samples ranged from 6.29 to 6.54 log CFU/g, respectively (Socaciu et al., 2021). Also, other studies have reported the effect of other plant essential oils (such as cinnamon and cumin) on the inhibition of lactic acid bacteria (Ghaderi‐Ghahfarokhi et al., 2017; Homayonpour et al., 2021).

### 3.6 Chemical analysis

**FIGURE 4:**
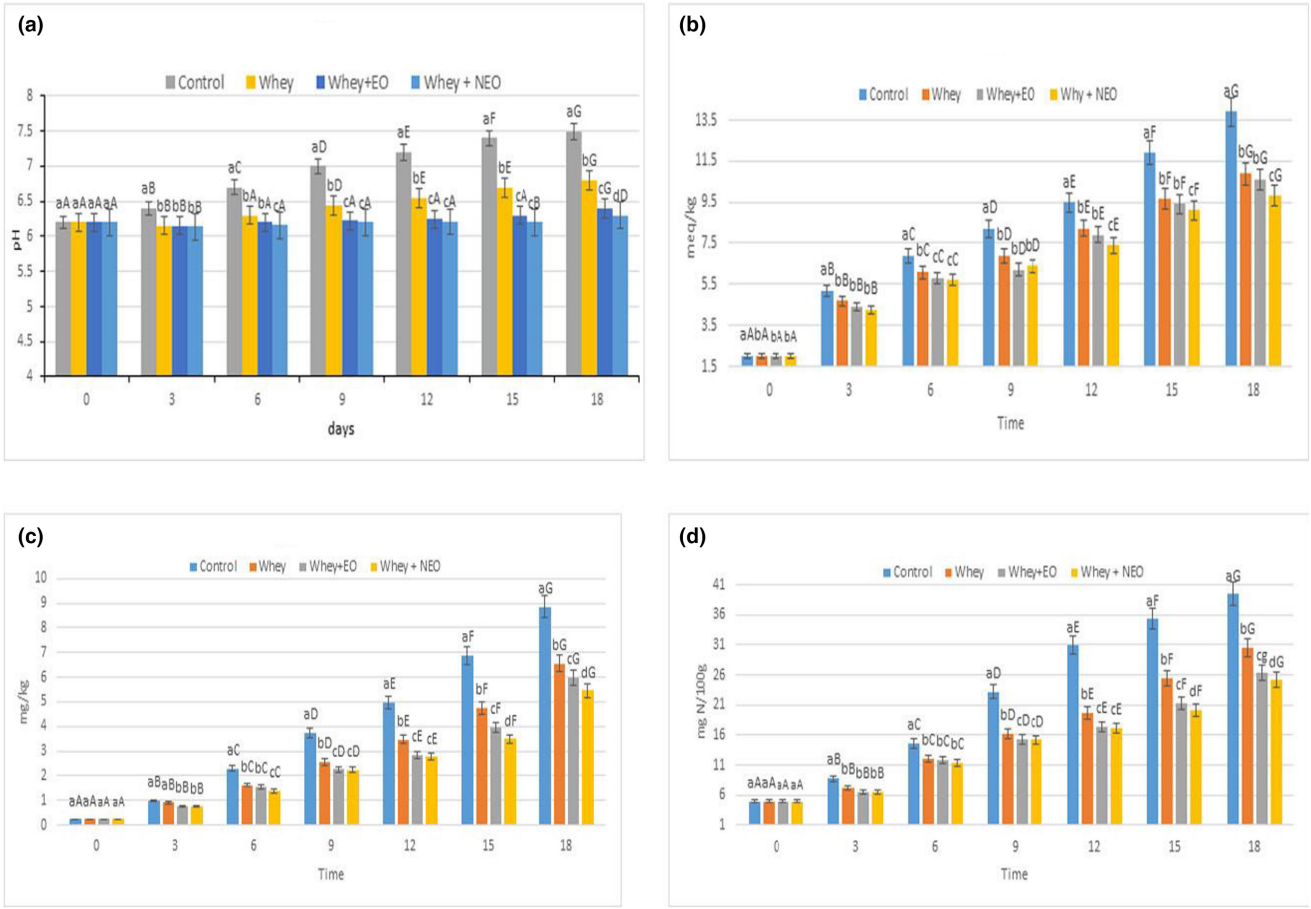
pH changes of control and treatments during 18 days (a), PV changes of control and treatments during 18 days (b), TBARs changes of control and treatments during 18 days (c), and TVB‐N changes of control and treatments during 18 days (d). In each group different lowercase letters are significantly different, *p* < .05; between group different superscript letters are significantly different, *p* < .05.

All chemical analyses (pH, PV, TBARS, and TVB‐N) are shown in Figure 4.

#### 3.6.1 pH value

Figure 4a shows there wasn't a difference on day zero between the all samples (pH = 6.2). After 3 days, the pH value was changed in the control and treatment samples. After 18 days, the minimum and maximum detected pH values were associated with whey‐NEO (pH = 6.3) and control samples (pH = 7.5), respectively. Also, the pH value after 18 days in whey and whey‐EO treatments was 6.8 and 6.4, respectively, somewhat similar to whey‐NEO treatment. Changes in the pH value of the treatments (forms EO and NEO) can be due to the growth of bacteria during storage. In addition, the increase in pH value in beef samples can be caused by the autolysis process and the production of alkaline substances (such as ammonia (NH_3_), indole (C_8_H_7_N), histamine (C_5_H_9_N_3_), and trimethylamine (C_3_H_9_N)). Endogenous activity (such as lipases and proteases) or enzymatic activity of microorganisms leads to the increase of volatile bases during storage. In a similar study, Zomorodian et al. investigated the impact of coatings containing chitosan and Shirazi thyme EO (forms of Pickering emulsion and free) on the amount of pH value of salmon fish and stated that the amount of pH in Pickering treatment was the lowest (pH = 6.84 after 16 days of storage) compared to the control sample (Zomorodian et al., 2023). Also, Socaciu et al. (analyzing the effects of coatings containing tarragon essential oil and whey) stated that the pH values for control and treatment samples varied from 6.5 to 6.7, respectively, after 15 days (Socaciu et al., 2021).

#### 3.6.2 PV value

Figure 4b shows the PV value changes over 18 days for the control and treatment samples. There was no difference in the amount of PV value on day zero (2 meq/kg). After 3 days, the change in PV value was observed in the control and treatment samples. After 18 days, the maximum and minimum PV values were associated with the control sample (13.9 meq/kg) and whey‐NEO treatment (9.8 meq/kg), respectively. Also, after 18 days, the PV value of whey and whey‐EO was 10.9 and 10.6 meq/kg, respectively. The PV changes in the treatments (forms of EO and NEO) could be due to the substances of flavonoid and phenolic release in the beef samples in storing time. Furthermore, during meat storage, the quantity of PV value enhanced significantly in the control and treatments, which resulted from the formation of fatty acids (short‐chain) due to the bacterial enzymes hydrolysis, which is sensitive to the possess of oxidation and conducted to the peroxide formation (Aşik & Candoğan, 2014; Homayounpour et al., 2021; Kizil et al., 2010; Pabast et al., 2018). In a similar study, Zomorodian et al. studied the effect of coating consisting of *Z. multiflora* EO (forms of Pickering emulsion and free) and chitosan on the amount of PV of salmon fish and stated that the amount of PV value in Pickering emulsion treatment was the lowest (0.72 meq/kg after 16 days of storage) among other treatments and control samples (Zomorodian et al., 2023). Also, Boghori et al. analyzed the effect of coating containing *Z. multiflora* EO and whey and expressed that the PV value of treatment and control samples after 12 days varied from 1.31 to 4.22 meq/kg, respectively (Boghori et al., 2020). Our results were also comparable to the other research with other EOs (Homayounpour et al., 2020; Shariatifar et al., 2019; Tometri et al., 2020).

#### 3.6.3 TBARs value

Figure 4c shows the changes in TBARs during 18 days in control and treatment samples. Based on this figure, no difference was observed in the amount of TBARs on day zero (0.23 mg MDA/kg). After 3 days, a change in the amount of TBARs was observed in the control and treatment samples. Our results showed that the minimum and maximum amount of TBARs after 18 days were related to whey‐NEO (5.45 mg MDA/kg) and control (8.85 mg MDA/kg), respectively, and that on this day, the TBARs values of whey and whey‐EO were 6.55 and 5.97 mg MDA/kg, respectively. The reduction of TBARs in the treatment of whey‐NEO is possibly due to the controlled release of EO on the beef exterior layer. In beef samples, lipid peroxidation can occur by auto‐oxidation and photo‐sensitized oxidation or with the occurrence of an enzymatic reaction, like those relating to peroxidase and lipoxygenase, and enzymes of bacteria (Aşik & Candoğan, 2014; Homayounpour et al., 2021; Kizil et al., 2010; Pabast et al., 2018). In a similar study, Zomorodian et al. examined the effect of coating containing *Z. multiflora* EO (forms of Pickering emulsion and free) and chitosan on the TBARs content of salmon fish and stated that the amount of TBARs value in Pickering emulation treatment was the lowest (1.17 mg MDA/kg after 16‐day storage) compared to the control sample (Zomorodian et al., 2023). Furthermore, in the research of Socaciu et al. examined the effects of coatings containing tarragon essential oil and whey, they showed that the value of TBARs in treatment and control samples after 15 days ranged from 0.75 to 0.83 mg MDA/kg, respectively (Socaciu et al., 2021). Additionally, the findings of the present research were similar to the research of (Rahimabadi et al. (2012) and Homayonpour et al. (2021)), which investigated the impact of other essential oils such as Shirazi thyme and cumin.

#### 3.6.4 TVB‐N value

The changes of TVB‐N in the control and treatment samples during 18 days of storage can be seen in Figure 4d. Based on this figure, no difference in TVB‐N content was observed on day zero (5 mg N/100 g). After 3 days, the change of TVB‐N value was observed in the control and treatment samples. As Figure 4d shows, the minimum and maximum amount of TVB‐N after 18 days were related to whey‐NEO treatment (25.1 mg N/100 g) and control samples (39.4 mg N/100 g), respectively. TVB‐N in whey and whey‐EO treatments were 30.5 and 26.3 mg N/100 g, respectively. The change in TVB‐N amount is due to protein degradation, enzymes, derivatives in beef, and various volatile bases produced, such as trimethylamine (C_3_H_9_N), ammonia (NH_3_), indole (C_8_H_7_N), and histamine (C_5_H_9_N_3_) (Tometri et al., 2020). In a similar study, Zomorodian et al. evaluated the effect of coating containing *Z. multiflora* EO (forms of Pickering emulsion and free) and chitosan on the amount of TVB‐N of salmon fish and stated that the amount of TVB‐N value in Pickering treatment (nano form) was the lowest (43.5 mg N/100 g after 16 days of storage), among other treatments and control samples (Zomorodian et al., 2023). Furthermore, in the research of Socaciu et al., the effects of coating containing tarragon EO and whey were researched; they showed that the amount of TVB‐N after 15 days for treatment and control samples was varied from 3.64 to 4.55 mg N/100 g, respectively (Socaciu et al., 2021). Additionally, the outcomes of the present investigation were similar to the research of Tometri et al., who expressed that compared to the free form of *Laurus nobilis* leaf extract, the nanoform had a better impact (Tometri et al., 2020).

### 3.7 Sensory evaluation

Based on Table 2, sensory evaluation of control and treatment samples can be seen during 18 days of storage. Regarding meat texture, after 12 days of storage, the control samples scored 4, which is unacceptable (above 3 was unacceptable), but the treatment samples scored less than 3 during 18 days, which is acceptable. In this case, the best quality (the lowest score) was related to the whey‐NEO (score = 1). Regarding the off‐odor parameter of meat samples, the control samples obtained a score of 4, which is unacceptable, after 9 days, and the whey treatment obtained this score after 15 days. Considering the other treatments (whey‐EO and whey‐NEO), they received an acceptable score during the entire testing period. The lowest score or the highest quality on the 18th day was related to the whey‐NEO (score = 1). Regarding the parameter of discoloration in meat samples, the control and whey samples received an unacceptable score after 12 days (score = 4), and the other treatments (whey‐EO and whey‐NEO) received an acceptable score during the 18 days of testing. Like the previous two parameters, the best quality or the lowest score was related to the whey‐NEO sample after 18 days of storage (score = 1). Concerning the red color parameter in meat samples, the control sample after 12 days and the whey treatment after 15 days scored an unacceptable scale (score = 4), and the other treatments (whey‐EO and whey‐NEO) obtained an acceptable score during the 18 days of testing. Regarding this parameter, the best quality or the lowest score after 18 days of storage was related to the whey‐NEO (score = 1). As expressed in the 4 parameters of sensory evaluation, EO treatments (free and Pickering emulsion form) had the best quality during 18 days of storage, which is possibly owing to the properties of the essential oil and its combination with whey, that the Pickering emulsion (whey‐NEO) has shown more effect. These treatments effectively reduced the growth of microbials in meat samples and reduced the rate of protein/lipid degradation, as well as the accumulation of volatile compounds in meat (Mohan et al., 2012). In prior research conducted by Socaciu et al. (analyzed the effects of coatings containing tarragon EO and whey), they expressed that the sensory properties of treatments after 15 days were better than control samples (Socaciu et al., 2021). Also, based on another investigation, it has been expressed that the use of Pickering technology raises the quality of sensory items of foodstuffs (Bazargani‐Gilani et al., 2015). Furthermore, our outcomes were confirmed by the Zomorodian et al. study, which investigated the impact of coatings containing chitosan and Shirazi thyme EO (forms of Pickering emulsion and free) on the sensory items of salmon fish and stated that the best quality or lowest score were related to the Pickering treatment (nano form) among other treatments and the control sample (Zomorodian et al., 2023).

**TABLE 2:** Sensory evaluation in meat samples during storage (18 days storage at 4°C).

Parameters	Treatments	Days						
		0	3	6	9	12	15	18
Texture	Control	1	1	2	3	4	5	5
	Whey	1	1	1	1	2	2	2
	Whey+EO	1	1	1	1	1	2	2
	Whey + Nano EO	1	1	1	1	1	1	1
Off‐odor	Control	1	2	3	4	5	5	5
	Whey	1	1	2	2	3	4	5
	Whey+EO	1	1	1	1	1	2	3
	Whey + Nano EO	1	1	1	1	1	1	1
Discoloration	Control	1	1	2	3	4	5	5
	Whey	1	1	1	2	4	5	5
	Whey+EO	1	1	1	1	1	2	3
	Whey + Nano EO	1	1	1	1	1	1	1
Red color	Control	1	1	2	3	4	5	5
	Whey	1	1	1	2	3	4	5
	Whey+EO	1	1	1	1	1	2	3
	Whey + Nano EO	1	1	1	1	1	1	1

*Note*: A score of 1 means the highest quality and a score of 5 means the lowest quality. Also, when the sensory characteristics were higher than the value of three, the sample was no longer of good quality and was rejected.

## 4 CONCLUSION

In the present study, *Trachyspermum copticum L*. essential oil along with whey protein were used as an edible coating for beef preservation. SEM and TEM images confirmed the production of this edible coating loaded with emulsion. According to our findings, evaluations of chemical and microbial analyses revealed that the treatment of whey‐NEO led to a postponement in microbial and chemical spoilage and enhanced the shelf‐life of beef meat samples during storage. The essential oil in the form of Pickering emulsion permits the controlled release of bioactive agents on beef to enhance the activity of antimicrobials and antioxidants during 18 days of storage compared to the form of free EO. Results showed that the coating based on whey and NEO can be applied as a biodegradable and edible coating in beef preservation. One of the limitations of the current research was the existence of financial limitations to evaluate and compare the effect of the extract (aqueous and ethanolic) of this plant (*Trachyspermum copticum L*.) on beef characteristics, which is suggested to be investigated in the future.

## AUTHOR CONTRIBUTIONS

**Vida Saghari:** Conceptualization (equal); data curation (equal); funding acquisition (equal); investigation (equal); methodology (equal); resources (equal); software (equal); writing – original draft (equal). **Hossein Jalali:** Conceptualization (equal); methodology (equal); resources (equal); writing – review and editing (equal). **Nabi Shariatifar:** Resources (equal); software (equal); supervision (equal); validation (equal); visualization (equal); writing – review and editing (equal). **Seyedhamidreza Ziaolhagh:** Data curation (equal); formal analysis (equal); funding acquisition (equal); investigation (equal); methodology (equal); project administration (equal); writing – original draft (equal).

## FUNDING INFORMATION

No financial funds were used in this article.

## CONFLICT OF INTEREST STATEMENT

The authors have no relevant financial or non‐financial interests to disclose.

## ETHICS STATEMENT

This study does not involve any human or animal testing.

## Data Availability

The datasets used and/or analyzed during the current study are available from the corresponding author on reasonable request.
